# A Direct Georeferencing Method for Terrestrial Laser Scanning Using GNSS Data and the Vertical Deflection from Global Earth Gravity Models

**DOI:** 10.3390/s17071489

**Published:** 2017-06-24

**Authors:** Edward Osada, Krzysztof Sośnica, Andrzej Borkowski, Magdalena Owczarek-Wesołowska, Anna Gromczak

**Affiliations:** Institute of Geodesy and Geoinformatics, Wrocław University of Environmental and Life Sciences, ul, Grunwaldzka 53, 50-357 Wrocław, Poland; edward.osada@igig.up.wroc.pl (E.O.); krzysztof.sosnica@igig.up.wroc.pl (K.S.); magdalena.owczarek-wesolowska@igig.up.wroc.pl (M.O.-W.); anna.gromczak@gmail.com (A.G.)

**Keywords:** terrestrial laser scanning, georeferencing, reference frame transformation, GNSS positioning, spatial directional intersection, Earth gravity model, deflection of the vertical

## Abstract

Terrestrial laser scanning is an efficient technique in providing highly accurate point clouds for various geoscience applications. The point clouds have to be transformed to a well-defined reference frame, such as the global Geodetic Reference System 1980. The transformation to the geocentric coordinate frame is based on estimating seven Helmert parameters using several GNSS (Global Navigation Satellite System) referencing points. This paper proposes a method for direct point cloud georeferencing that provides coordinates in the geocentric frame. The proposed method employs the vertical deflection from an external global Earth gravity model and thus demands a minimum number of GNSS measurements. The proposed method can be helpful when the number of georeferencing GNSS points is limited, for instance in city corridors. It needs only two georeferencing points. The validation of the method in a field test reveals that the differences between the classical georefencing and the proposed method amount at maximum to 7 mm with the standard deviation of 8 mm for all of three coordinate components. The proposed method may serve as an alternative for the laser scanning data georeferencing, especially when the number of GNSS points is insufficient for classical methods.

## 1. Introduction

Terrestrial laser scanning is one of the most efficient methods for providing the highly accurate and dense point clouds that can subsequently be used for measuring Earth surface processes [1], monitoring the deformations caused by natural hazards [2,3] or generating three-dimensional spatial models, e.g., [4,5,6,7,8]. The point clouds collected by a terrestrial laser scanner require georeferencing [9,10], i.e., the collected points have to be transformed to a reference frame that has a well-defined origin, orientation and scale. The reference ellipsoids of the Geodetic Reference System 1980 (GRS80) [11] or the World Geodetic System 1984 (WGS84) are commonly used as the geocentric coordinate frames for georeferencing of spatial data, because, e.g., the GPS coordinates are directly measured and estimated in WGS84.

In terrestrial laser scanning, the point cloud is typically transformed into the geocentric coordinate frame using a 7-parameter Helmert transformation [6]. Three coordinate components of the laser scanner’s origin, the scale, and three orientation angles with respect to the geocentric reference frame are needed to perform such a transformation. The set of seven transformation parameters is determined using the least squares method on a basis of several georeferencing points whose coordinates are measured both by the laser scanner and a global navigation satellite system (GNSS) receiver. Alternatively, the optical head of a laser scanner may be equipped with several mounted GPS antennas [12,13,14]. Other methods of georeferencing of terrestrial scanner data comprise back-sighting, sensor-driven, and data-driven approaches [10,15] which also rely on control point targets.

In some cases, for instance in city corridors, the availability of control points determined by GNSS can be limited due to deteriorated GNSS signal. Therefore, additional terrestrial measurements may be needed in order to ensure the sufficient number of control points in such situations.

To avoid this possible additional effort, we propose a method for direct georeferencing of point clouds obtained using terrestrial laser scanning that allows for providing coordinates in the geocentric frame using a minimum number of GNSS measurements. In the proposed method, three coordinate components of the laser scanner’s measurement origin, two components describing the vertical deflection of the scanner’s rotational axis and one angle of the horizontal orientation of the scanner’s measurement reference frame provide a minimum set of parameters that are needed for georeferencing and are entirely sufficient for a transformation to the geocentric reference frame. To apply the proposed method, the scanner must be levelled. The transformation parameters are estimated using the least squares method on a basis of the geocentric coordinates of the laser scanner’s origin, the geocentric coordinates of one GNSS point and the components of the vertical deflection of the scanner’s rotational axis determined in the gravitational force field of the Earth Gravity Model 2008 (EGM2008) [16] which gives the components of axis deflection with respect to the reference ellipsoid GRS80. Furthermore, we show results from a field test that proves correctness of the results obtained using proposed method.

The paper is structured as follows: First, we introduce laser scanner geocentric orientation parameters, then an adjustment procedure for determination of the orientation parameters is described. In the following section, a field test of the proposed method is provided. Finally the method is discussed in an extended context and conclusions are drawn.

## 2. Methods

### 2.1. The Laser Scanner Geocentric Orientation Parameters

**Figure 1:**
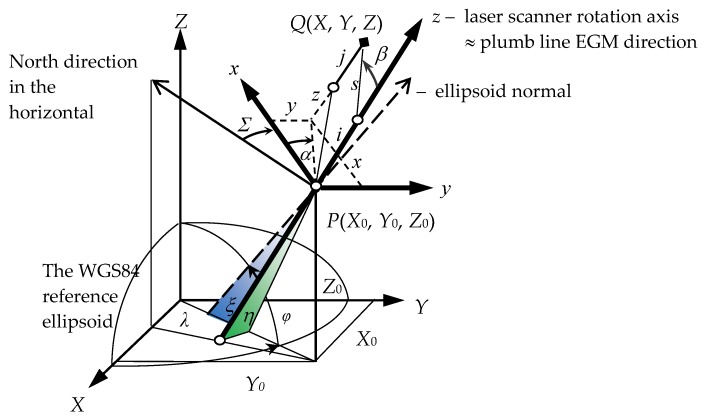
Six parameters of the laser scanner geocentric orientation: *X*_0_, *Y*_0_, *Z*_0_, *Σ*, *ξ*, *η.*

The external geocentric orientation parameters of the laser scanner’s measurement frame, assuming the equality of the scale in the both reference frames, reads as follows (see Figure 1):

- *X*_0_, *Y*_0_, *Z*_0_ geocentric GRS80 coordinates of the origin *P* of the laser scanner’s measurement frame (*x*, *y*, *z*),
- *Σ*, *ξ*, *η* orientation angles of the measuring frame (*x*, *y*, *z*) with respect to the external reference frame (*X*, *Y*, *Z*).

The *ξ*, *η* orientation angles constitute the components of the laser scanner vertical axis deflection with respect to the normal to the GRS80 reference ellipsoid (Figure 1), which is caused by the inhomogeneity of the local gravity field due to the local mass distribution. The directional horizontal angle *Σ* is called the instrument horizontal orientation constant.

The coordinates *x*, *y*, *z* of the point *Q* measured by a laser scanner can be obtained by (Figure 1):

(1) $$x=\left(\begin{array}{l} x\\ y\\ z \end{array}\right)=\left(\begin{array}{l} s\cos\alpha \sin\beta \\ s\sin\alpha \sin\beta \\ s\cos\beta +i-j \end{array}\right)$$

where *s*—spatial distance, *α*—horizontal direction, *β*—vertical angle (corrected due to refraction), *i*—height of the laser scanner above ground point *P*, *j*—height of the reflector above measured point *Q*, *j* = 0 for the point clouds and *j* > 0 for the georeferencing points. The origin of the laser scanner coordinate system (*x*, *y*, *z*) is usually defined as a point of the electro-optical center of the scanner or a point of the intersection of the horizontal and vertical rotation axes of the scanner or a zero distance measurement point of the scanner [17,18]. The connection of the computed vertical coordinate $\bar{z}=s\cos\beta -j$ with the *z* coordinate according to Formula (1) yields as follows: $z=\bar{z}+i$ (see Figure 1).

The coordinates (*x*, *y*, *z*) of a particular point *Q* measured by a laser scanner are converted into the (*X*, *Y*, *Z*) external reference frame according to the formula (e.g., [19,20]):

(2) $$\left(\begin{array}{l} X\\ Y\\ Z \end{array}\right)=\left(\begin{array}{l} X_{0}\\ Y_{0}\\ Z_{0} \end{array}\right)+{\left(R\left(\Sigma \right)\cdot Q\left(\xi ,\eta ,\varphi \right)\cdot P\left(\varphi ,\lambda \right)\right)}^{T}\left(\begin{array}{l} x\\ y\\ z \end{array}\right)$$

or equivalently:

(3) $$X=X_{0}+{\left(R\left(\Sigma \right)\cdot Q\left(\xi ,\eta ,\varphi \right)\cdot P\left(\varphi ,\lambda \right)\right)}^{T}x$$

where:

(4) $$P\left(\varphi ,\lambda \right)=\left(\begin{array}{ccc} -\sin\varphi \cos\lambda & -\sin\varphi \sin\lambda & \cos\varphi \\ -\sin\lambda & \cos\lambda & 0\\ \cos\varphi \cos\lambda & \cos\varphi \sin\lambda & \sin\varphi \end{array}\right)$$

(5) $$Q\left(\xi ,\eta ,\varphi \right)=\left(\begin{array}{ccc} 1 & -\eta \tan\varphi & -\xi \\ \eta \tan\varphi & 1 & -\eta \\ \xi & \eta & 1 \end{array}\right)$$

(6) $$R\left(\Sigma \right)=\left(\begin{array}{ccc} \cos\Sigma & \sin\Sigma & 0\\ -\sin\Sigma & \cos\Sigma & 0\\ 0 & 0 & 1 \end{array}\right)$$

with *ϕ*, *λ* being the geodetic latitude and longitude of the laser scanner’s or total station’s reference frame origin *P*.

Many authors, e.g., [19,20,21,22] use a set of three alternative angles for georeferencing: *Σ*, *Φ* and *Λ*, where *Φ* denotes the astronomical latitude and *Λ* denotes the astronomical longitude. This paper’s orientation parameters (*ξ*, *η*) and (*Φ*, *Λ*) are connected by well-known relations [22]:

(7) ξ=Φ−φ,

(8) η=(Λ−λ)cosφ.

Five laser scanner orientation parameters (*X*_0_, *Y*_0_, *Z*_0_, *ξ*, *η*) can be obtained by GNSS measurements providing the coordinates of the origin (*X*_0_, *Y*_0_, *Z*_0_) and the computation of two orientation angles (*ξ*, *η*) on a basis of the global Earth gravity model [23]. The sixth parameter *Σ* can be obtained by solving Equation (2) for given laser scanner measurements (*s*, *α*, *β*, *i*, *j*) and GNSS measurements of the coordinates of one georeference point *Q*(*X*, *Y*, *Z*) (see Figure 1).

Finally, the coordinates *x*, *y*, *z* of the point clouds measured by a laser scanner can be directly transformed to the geocentric coordinates according to the Equation (2) in which the computed values of six orientation parameters *X*_0_, *Y*_0_, *Z*_0_, *ξ*, *η*, *Σ* are used. Prior to the transformation, the orientation parameters can be adjusted and thus also improved by the least squares method which provides the expected values of estimated parameters as well as standard deviations of estimated parameters and adjusted observations.

### 2.2. Adjustment of the Laser Scanner Geocentric Orientation

In the proposed procedure, the laser scanner orientation parameters (*X*_0_, *Y*_0_, *Z*_0_, *ξ, η*, *Σ*) computed on the basis of the laser scanner measurements in the local frame (*x*, *y*, *z*), the GNSS measurements in the global frame (*X*_0_, *Y*_0_, *Z*_0_, *X*, *Y*, *Z*) and EGM2008 vertical deflection data (*ξ*, *η*) can be integrated and improved using the common least squares adjustment including the a priori covariance information of adjusted parameters and observations.

The nonlinear observational equation of the integrated laser scanner, GNSS, and EGM2008 data based on the Equation (2) reads as follows:

(9) $$\left(\begin{array}{l} X+v_{X}\\ Y+v_{Y}\\ Z+v_{Z} \end{array}\right)=\left(\begin{array}{l} X_{0}+v_{X_{0}}\\ Y_{0}+v_{Y_{0}}\\ Z_{0}+v_{Z_{0}} \end{array}\right)+{\left(R\left(\Sigma +d\Sigma \right)\cdot Q\left(\xi +v_{\xi },\eta +v_{\eta },\varphi \right)\cdot P\left(\varphi ,\lambda \right)\right)}^{T}\left(\begin{array}{l} x+v_{x}\\ y+v_{y}\\ z+v_{z} \end{array}\right)$$

or equivalently

(10) $$X+v_{X}=X_{0}+v_{X_{0}}+{\left(R\left(\Sigma +d\Sigma \right)\cdot Q\left(\xi +v_{\xi },\eta +v_{\eta },\varphi \right)\cdot P\left(\varphi ,\lambda \right)\right)}^{T}(x+v_{x})$$

where $v_{X},v_{Y},v_{Z},v_{X_{0}},v_{Y_{0}},v_{Z_{0}}$—random errors of the GNSS measured coordinates $X,\;Y,\;Z,\;X_{0},\;Y_{0},\;Z_{0}$ of the points *P* and *Q*; $v_{x},v_{y},v_{z}$—random errors of the laser scanner measured coordinates x, y, z of the point *Q*; *v_ξ_*, *v_η_*—random errors of the deflection of the vertical components *ξ*, *η*, *dΣ*—correction of the approximated value of the horizontal orientation angle *Σ.*

The nonlinear observational Equation (10) can easily be linearized using the expansion into the Taylor series and we receive the Gauss–Helmert model (comp. e.g., [24]):

(11) Bv+AdΣ+w=0

where

(12) $$v={\left[v_{x},v_{y},v_{z},v_{X_{0}},v_{Y_{0}},v_{Z_{0}},v_{X},v_{Y},v_{Z},v_{\xi },v_{\eta }\right]}^{T}$$

(13) $$w=X-X_{0}-{\left(R\left(\Sigma \right)\cdot Q\left(\xi ,\eta ,\varphi \right)\cdot P\left(\varphi ,\lambda \right)\right)}^{T}x$$

(14) $$A=-{\left(\frac{dR\left(\Sigma \right)}{d\Sigma }\cdot Q\left(\xi ,\eta ,\varphi \right)\cdot P\left(\varphi ,\lambda \right)\right)}^{T}x$$

(15) $$B=\left[\begin{array}{c} -{\left(R\left(\Sigma \right)\cdot Q\left(\xi ,\eta ,\varphi \right)\cdot P\left(\varphi ,\lambda \right)\right)}^{T}\\ -I\\ I\\ -{\left(R\left(\Sigma \right)\cdot \frac{dQ\left(\xi ,\eta ,\varphi \right)}{d\xi }\cdot P\left(\varphi ,\lambda \right)\right)}^{T}\\ -{\left(R\left(\Sigma \right)\cdot \frac{dQ\left(\xi ,\eta ,\varphi \right)}{d\eta }\cdot P\left(\varphi ,\lambda \right)\right)}^{T} \end{array}\right]$$

(16) $$Ι=\left[\begin{array}{ccc} 1 & & \\ & 1 & \\ & & 1 \end{array}\right]$$

(17) $$\frac{\partial R\left(\Sigma \right)}{\partial \Sigma }=\left[\begin{array}{ccc} -\sin\varphi & \cos\varphi & 0\\ -\cos\varphi & -\sin\varphi & 0\\ 0 & 0 & 0 \end{array}\right]$$

(18) $$\frac{\partial Q\left(\xi ,\eta ,\varphi \right)}{d\xi }=\left[\begin{array}{ccc} 0 & 0 & -1\\ 0 & 0 & 0\\ 1 & 0 & 0 \end{array}\right]$$

(19) $$\frac{\partial Q\left(\xi ,\eta ,\varphi \right)}{d\eta }=\left[\begin{array}{ccc} 0 & -\tan\varphi & 0\\ \tan\varphi & 0 & -1\\ 0 & 1 & 0 \end{array}\right]$$

The linear observational equation of the integrated laser scanner, GNSS, and EGM data (11) can be solved using the weighted least squares method:

(20) $$v^{T}Pv=\min$$

where $P=\Sigma_{l}^{-1}$ is the weight matrix of the observation vector $l={\left[x,y,z,X_{0},X_{0},X_{0},X,Y,Z,\;\xi ,\eta \right]}^{T}$ and $\Sigma_{l}=diag[\sigma_{x}^{2},\sigma_{y}^{2},\sigma_{z}^{2},\sigma_{X_{0}}^{2},\sigma_{Y_{0}}^{2},\sigma_{Z_{0}}^{2},\sigma_{X}^{2},\sigma_{Y}^{2},\sigma_{Z}^{2},\;\sigma_{\xi }^{2},\sigma_{\eta }^{2}]$ is the diagonal covariance matrix with the squares of standard deviations of the observations. In general, the covariance matrix $\Sigma_{l}$ is not diagonal but includes known covariances between coordinates derived by GNSS observations $\left(\sigma_{X_{0}Y_{0}},\sigma_{X_{0}Z_{0}},\sigma_{Y_{0}Z_{0}},\sigma_{XY},\sigma_{XZ},\sigma_{YZ}\right)$, and covariances between coordinates obtained by laser scanner measurements $\left(\sigma_{xy},\sigma_{xz},\sigma_{yz}\right)$. The variances $\left(\sigma_{x}^{2},\sigma_{y}^{2},\sigma_{z}^{2}\right)$ and covariances $\left(\sigma_{xy},\sigma_{xz},\sigma_{yz}\right)$ are computed on the basis of Equation (1) using the variance-covariance propagation law formulae, including known standard deviations of the uncorrelated (independent) observations $\left(\sigma_{s},\sigma_{\alpha },\sigma_{\beta },\sigma_{i},\sigma_{j}\right)$.

The solution of the observational Equation (11) under the condition (20) is given by (e.g., [25]):

(21) $$d\Sigma =-{\left(A^{T}M^{-1}A\right)}^{-1}A^{T}M^{-1}w$$

(22) $$v=-P^{-1}B^{T}M^{-1}(Ad\Sigma +w)$$

where $M=BP^{-1}B^{T}$.

The adjusted parameter $\hat{\Sigma }$ and the vector of adjusted observations $\hat{l}$ read as:

(23) $$\hat{\Sigma }=\Sigma_{0}+d\Sigma$$

(24) $$\hat{l}=l+v$$

The variance of the adjusted parameters $\hat{\Sigma }$, the covariance matrix of the residual vector **v** and the covariance matrix of adjusted observations **l***_a_* can be expressed as:

(25) $$\sigma_{\hat{\Sigma }}^{2}={\left(A^{T}M^{-1}A\right)}^{-1}$$

(26) $$\Sigma_{v}=-P^{-1}B^{T}M^{-1}[M-A{\left(A^{T}M^{-1}A\right)}^{-1}A^{T}]M^{-1}BP^{-1}$$

(27) $$\Sigma_{\hat{l}}=\Sigma_{l}-\Sigma_{v}$$

Finally, the coordinates *x*, *y*, *z* of the point cloud measured by a laser scanner are directly converted to the geocentric coordinates according to the Equation (2) where the adjusted values of six orientation parameters yield the sum of the a priori values of parameters and the improvements of parameters obtained from the least squares adjustment: ${\hat{X}}_{0}=X_{0}+v_{X_{0}}$, ${\hat{Y}}_{0}=Y_{0}+v_{Y_{0}}$, ${\hat{Z}}_{0}=Z_{0}+v_{Z_{0}}$, $\hat{\xi }=\xi +v_{\xi }$, $\hat{\eta }=\eta +v_{\eta }$, $\hat{\Sigma }=\Sigma_{0}+d\Sigma$.

## 3. Results of the Field Experiment

**Figure 2:**
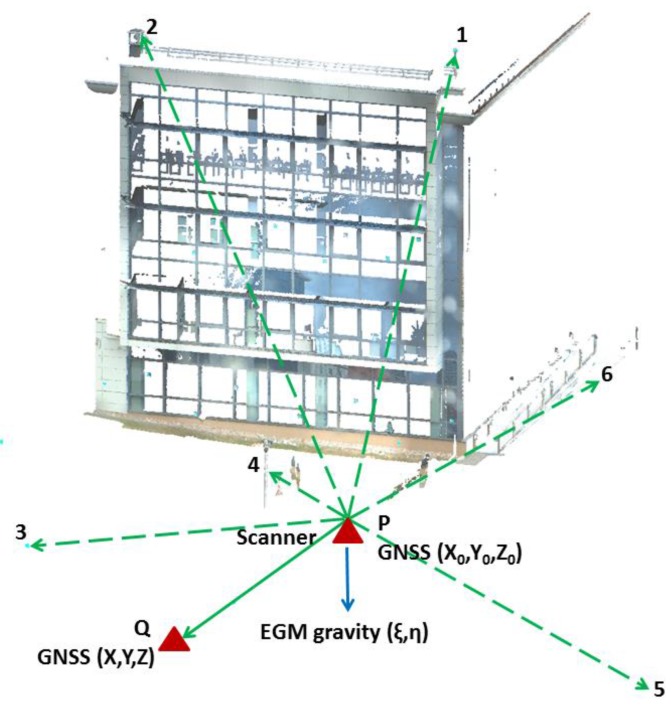
The data used in the field experiment–a part of the point cloud of a building façade.

The proposed method was validated using real GNSS and laser scanning measurements obtained in the framework of a field experiment. The position of the laser scanner and the position of the GNSS point used in the experiment are shown in Figure 2. *P*(*X*_0_, *Y*_0_, *Z*_0_) denotes the laser scanner reference point position measured by a GNSS receiver; *Q*(*X*, *Y*, *Z*) is the georeferencing point measured both by the laser scanner and the GNSS receiver; EGM gravity denotes the direction of the plumb line from the global Earth gravity model EGM2008, assuming that the plumb line direction coincides with the vertical laser scanner rotation axis. Points 1, 2, 3, 4, 5 and 6 are remote testing points extracted from the point cloud and measured by the GNSS receiver, as well. The testing points 1 and 2 are located on the building’s roof, 20 m over the scanner’s position. The remaining points are located on the ground. In order to ensure unambiguous identification of the testing points in the point cloud, the identification targets were placed on both the roof points and on the ground points while scanning. The typical vendor’s targets were used as the identification targets and also scanner manufacturer software (Leica Cyclone) was used in order to determine the target center.

The following data are considered in the field experiment:

- **Laser scanner data**: The point cloud coordinates {*x*, *y*, *z*} of the testing points 1, 2, 3, 4, 5, 6 and of the georeferencing point *Q* are measured in the local reference frame of the laser scanner at the position *P* using laser scanner Leica P20. The heights of the laser scanner (*i*) and reflector (*j*) are also the measured quantities (see Table 1). The standard deviations, provided in Table 1, are the a priori values used for parameter estimation. These values were set up according to the Leica P20’s technical specifications.
- **GNSS data**: The *X*, *Y*, *Z* coordinates of the laser scanner position *P* and the georeferencing point *Q*, as well as the testing points on the roof 1, 2 and in the ground 3, 4, 5, 6 measured by GNSS (see Table 2). The GNSS coordinates of the testing points 1, 2, 3, 4, 5, 6 are not included in the least squares adjustment of the laser scanner position, as they are only used for the assessment of the laser scanner geocentric georeferencing accuracy. For the GNSS measurements, the GNSS Leica Viva GS08plus Smart Antenna receiver was used. The real-time kinematic (RTK) GNSS measurements were carried out using corrections from a single base station WROC (permanent station located in Wroclaw, Poland), provided by the ASG-EUPOS reference GNSS system in Poland [26]. WROC belongs to the global network of the International GNSS Service as well (IGS, [27]). The distance of the mobile GNSS receiver from the reference station WROC was shorter than 100 m. Each point was measured three times in 2 min sessions. The values provided in Table 2 are the mean values.
- **Global gravity field model data**: We used the northern *ξ* and the eastern *η* components of the vertical axis deflection of the laser scanner with respect to the normal to the GRS80 ellipsoid, which are calculated at the point *P* on the basis of the EGM2008 gravity field model. These values are calculated according to equations (7) and (8) where φ and λ are known from the GNSS positioning and
  (28) $$\Phi =\arctan((-\partial W/\partial Z)/\sqrt{{\left(\partial W/\partial X\right)}^{2}+{\left(\partial W/\partial Y\right)}^{2}},$$
  (29) Λ=arctan((∂W/∂Y)/(∂W/∂X).

For the practical applications in the lowlands, the components of the vertical deflections can be easily interpolated from the global vertical deflection (GVD) model provided on the internet e.g., by the National Geospatial-Intelligence Agency [28]. However, the GVD model is related to the surface of the geoid, whereas the measurements are performed on the Earth’s surface. Thus, such an approximation may be applied only to the lowland areas, where the differences between vertical deflections on the geoid and Earth surface are negligible. For mountainous areas, Equations (28) and (29) should be used for deriving correct values of the vertical deflections.

The components of the vertical deflection, calculated using EGM2008, amount to *ξ* = 5.99, *η* = 6.20 in arc sec. According to producers of laser scanners, the direction of the vertical axis is consistent with the plumb line direction to the order of 1 arcsec. Therefore, in the adjustment process, the standard deviations of the components of the vertical deflection are assumed *σ_ξ_* = *σ_η_* = 1 arcsec. The EGM2008 gravity has been selected as one of the most highly accurate global gravity field models, which is based on a combination of satellite laser ranging data for the longest wavelengths of the gravity field, the Gravity Recovery and Climate Experiment (GRACE) data for the long and mid wavelengths of the gravity field, as well as from the altimetry and terrestrial measurements for the local gravity. Pavlis et al. [16] show that EGM2008 provides very accurate gravity anomaly, disturbance, and vertical deflection data from global to local scales and the model is particularly well-suited for the area of Central Europe with the RMS of differences to the local geoid, e.g., in Germany at a level of 3.0–3.5 cm.

The approximated value of the parameter *Σ* equals to 305.8411 grad. The correction to the orientation angle *Σ* is computed solving Equation (2) for given laser scanner measurements (*s*, *α*, *β*, *i*, *j*) and GNSS measurements of the coordinates of the laser scanner point *P*(*X*_0_, *Y*_0_, *Z*_0_) and the georeferencing point *Q*(*X*, *Y*, *Z*), using the Levenberg–Marquardt method of conjugate gradients. The obtained adjusted corrections $v_{x}$ $v_{y}$ $v_{z}$ $v_{X_{0}}$ $v_{Y_{0}}$ $v_{Z_{0}}$ $v_{X}$ $v_{Y}$ $v_{Z}$ $v_{\xi }$ $v_{\eta }$ of the parameters *x*, *y*, *z*, *X_0_*, *Y_0_*, *Z_0_*, *X*, *Y*, *Z*, *ξ*, *η* are acceptable when comparing to the standard deviations thereof, since the condition $\left|v\right|\leq 2\sigma_{v}$ is fulfilled for all cases.

Finally, the coordinates *x*, *y*, *z* of the point cloud measured by laser scanner are transformed to the geocentric coordinates according to Formula (2) with applying the adjusted values of six orientation parameters ${\hat{X}}_{0}$, ${\hat{Y}}_{0}$, ${\hat{Z}}_{0}$, $\hat{\xi }$, $\hat{\eta }$, $\hat{\Sigma }$. The accuracy of this transformation is tested by comparing the transformed geocentric coordinates of the test points 1, 2,…, 6 with the corresponding coordinates obtained by the GNSS receiver. Table 3 shows that all the differences of the coordinates measured by GNSS and from the transformation using the vertical deflection are below or at the level of 1 cm for the all test points.

Assuming that the accuracy of the *X*, *Y*, *Z* coordinate determination by the laser scanner depends on only GNSS positioning (which is not true, because additional errors come from scanning technology), the accuracy of coordinate differences can easily be calculated. Due to error propagation, it yields: 8$\sqrt{2}=11.3$ mm. This is the most optimistic evaluation. In fact, this error value could be significantly greater. Thus the coordinate differences summarized in Table 3 are in the range of their errors.

## 4. Discussion

As opposed to the classical georeferencing methods of point clouds, the proposed solution introduces the a priori information on two orientation angles of the measurement reference frame. Therefore, the geometry of the observations is directly linked to the local gravity field which gives the possibility of transforming the coordinates to the global geocentric reference frame. The link between the geometry and the gravity field is defined by two vertical deflection components of the scanner’s rotational axis that are determined from the global Earth gravity model. The proposed method requires measuring the geocentric coordinates of only two GNSS points: one point of the laser scanner’s position and one orientation point. In the classical solution, there is no a priori information on the gravitational field, and thus, the orientation of all three axes must be provided by geocentric coordinates, which is why at least three georeferencing GNSS points are needed. The proposed method allows for reducing the number of required GNSS georeferencing points to two through a direct link between the geometry provided by GNSS in the global reference frame, and terrestrial scanning measurements in the local reference frame, and the gravity information from the global Earth gravity model EGM2008, which provides the connection between the local and the global reference frames. In particular, the proposed method can be useful when the number of georeferencing GNSS points is limited or the accuracy of GNSS reference point positions have deteriorated due to poor satellite visibility, e.g., in the proximity of high buildings. It is typical that the GPS satellite visibility may be poor in city corridors. However, it is possible to find single gaps with a proper satellite configuration that can allow for georeferencing. Our method needs only two GNSS points with a proper determined position.

The advantage of the proposed method is more evident in case of terrestrial laser scanning performed indoor and outdoor of a building, e.g., for BIM (Buildings Information Model). In such a case, the indoor point cloud can be joined with the outdoor point cloud via one georeferencing point located outside the building. The scanner position inside the building can be determined when applying the method proposed in [29].

The integration of the vertical deflection with the classical method for georeferencing can also be used for the validation of the GNSS coordinates when any of the GNSS points are suspected to contain outliers or substantial systematic effects due to poor satellite observability or multipath. Even if the number of GNSS referencing points is small, the outliers or points affected by systematic errors can be successfully detected by a constraint derived from the orientation of the vertical axis in the local gravity field using the subsequent formulae.

The incorporation of the geometrical observations and the global gravitational field constitutes an important issue in the framework of the integration of precise surveying techniques.

The performed field experiment shows that the integration of the vertical deflection components from the global gravity Earth model together with geometric GNSS and laser scanning measurements leads to appropriate results in terms of the direct geocentric point cloud georeferencing, with the accuracy of less than 1 cm in terms of both the standard deviation and absolute differences. The proposed method of direct georeferencing can thus be used for the modelling of 3D objects in geocentric coordinate frames due to its accuracy, even when using a minimum number, i.e., merely two, of measured GNSS reference points.

**Figure 3:**
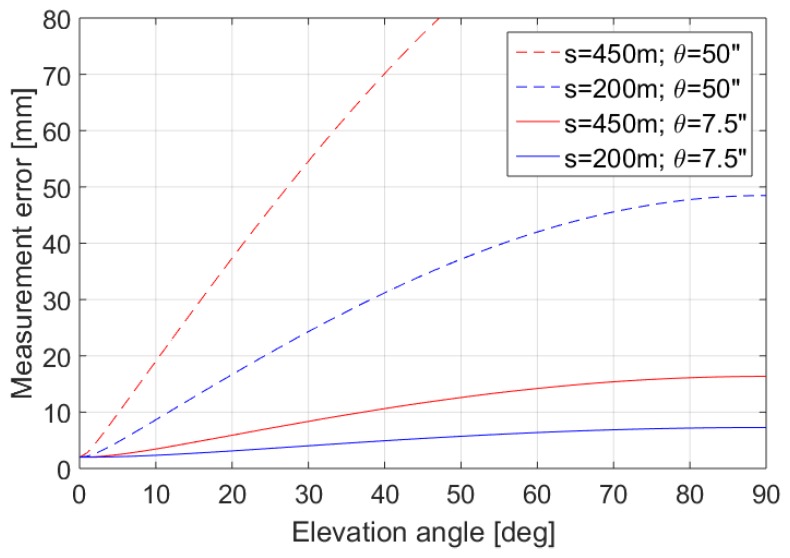
Measurement errors due to neglecting the vertical deflection in georeferencing for selected values of the vertical deflection *θ* = (*ξ*^2^ *+ η*^2^)^0.5^ in lowlands *θ* = 7.5” and mountainous areas *θ* = 50” and different distances *s* as a function of the elevation angle. Errors are calculated using Equation (1) and the error propagation law assuming the error of distance measurements *m_s_* = 2 mm.

The proposed method can be further simplified by neglecting the vertical deflections and thus assuming that the rotational axis of the laser scanner coincides with the normal to the ellipsoid. A similar approach using only two GNSS receivers for georeferencing and neglecting the vertical deflection was proposed by [12]. However, the neglect of the vertical deflection may introduce systematic effects in the estimated coordinates, which become substantial for long distances or for the mountainous areas, where the vertical deflections assume the largest values, which is illustrated in Figure 3. For the lowland areas with small vertical deflections, the measurement errors can assume values between 8 and 17 mm while scanning with high elevation angles. The measurement errors can increase up to 40–50 mm in the mountainous regions (blue dotted line in Figure 3) for the distances of 200 m, or may even exceed the value of 100 mm for the distance of 450 m. This consideration justifies the need of using the vertical deflection values. In fact, this is a component of the overall error budget, which in total is significantly greater, for example, due to the increase in the laser beam footprint when increasing the range of scanning.

Thus, using a full transformation with a proper and exact consideration of the vertical deflection as described in this paper is recommended for highly accurate georeferencing results to ensure that the results are free of any systematic errors.

## 5. Conclusions

In this paper, the proposed method is a tool for direct point cloud georeferencing in the global geocentric frame. The method relay on the combination of geometric information provided by GNSS and physical information provided by global gravity field model EGM2008 and is featured by:

- minimum number of GNSS measurements; only two reference points have to be positioned by GNSS,
- determination of vertical deflections component *ξ* and *η* as the scanner orientation parameters; *ξ* and *η* can be easily interpolated from a global vertical deflection model based on EGM2008.

Typically several GNSS points are used for georeferencing. However, it may turn out that some of those points must be excluded and thus cannot be used for georeferencing due to an inferior quality of their positions. Such a situation may happen in cities where the GNSS receivers are surrounded by very high buildings. The method proposed in this paper uses a minimum number of measured GNSS points. Thus, when other georeferencing methods fail due to an insufficient number of GNSS referencing points, the solution may still be possible assuming that we measure two GNSS point positions and we use vertical deflection values from EGM2008.

The conducted field test demonstrated that the method leads to the georeferencing accuracy at a level of a few millimeters. The proposed method can be deployed on sites where the GNSS signal may be deteriorated, e.g., in city corridors. Moreover, the method can be used in mountainous regions, where the vertical deflections assume the largest values and should not be neglected in order to capture high-accuracy point clouds.

Finally, by introducing the proposed method, relying not only on a minimum number of scanner targets, but also on the local gravity, it is possible to increase the speed and efficiency of point cloud acquisition and to independently verify whether the point cloud has proper georeferencing with respect to a global reference frame, such as the Geodetic Reference System 1980. Therefore, the proposed method, based on global scanner georeferencing, is a means for the reduction of necessary field measurements.

## Figures and Tables

**Table 1:** The results of the terrestrial laser scanning in the scanner’s local reference frame.

	Measured Values				
	*x* (m)	*y* (m)	*z* (m)	i (m)	j (m)
Georefer. point *Q*	−13.480	3.881	−0.076	1.843	1.763
Testing point: 1	18.612	−8.379	19.041	1.843	2.065
2	11.934	−22.744	19.045	1.843	1.563
3	−12.620	−10.105	0.086	1.843	1.862
4	6.315	−9.557	−0.014	1.843	1.567
5	−8.502	30.867	0.117	1.843	1.767
6	34.530	−8.366	0.206	1.843	1.862
Std. deviation	0.005	0.005	0.005	0.002	0.002

**Table 2:** The results of the global navigation satellite system (GNSS) field measurements in the global reference frame GRS80 in (m).

	Measured Values					
	X	St. Dev.	Y	St. Dev.	Z	St. Dev.
Laser scanner point *P*	3835659.499	0.008	1177290.998	0.008	4941636.307	0.008
Georefer. point *Q*	3835653.453	0.008	1177303.563	0.008	4941637.903	0.008
Testing point: 1	3835681.535	0.008	1177277.573	0.008	4941646.969	0.008
2	3835691.060	0.008	1177286.086	0.008	4941637.608	0.008
3	3835664.478	0.008	1177304.702	0.008	4941629.347	0.008
4	3835668.242	0.008	1177286.195	0.008	4941630.683	0.008
5	3835633.954	0.008	1177294.966	0.008	4941655.200	0.008
6	3835673.791	0.008	1177258.615	0.008	4941633.229	0.008

**Table 3:** Differences between geocentric converted and measured coordinates of the test points of the point cloud.

**Coordinates**	**Test Point 1**			**Test Point 2**		
	**GNSS Measured**	**Laser Scanner Transformed**	**Differences**	**GNSS Measured**	**Laser Scanner Transformed**	**Differences**
X (m)	3835681.535	3835681.530	−0.005	3835691.060	3835691.067	0.007
Y (m)	1177277.573	1177277.574	0.001	1177286.086	1177286.082	−0.004
Z (m)	4941646.969	4941646.964	−0.005	4941637.608	4941637.603	−0.005
**Coordinates**	**Test Point 3**			**Test Point 4**		
	**GNSS Measured**	**Laser Scanner transformed**	**Differences**	**GNSS Measured**	**Laser Scanner Transformed**	**Differences**
X (m)	3835664.478	3835664.482	0.004	3835668.242	3835668.244	0.003
Y (m)	1177304.702	1177304.709	0.007	1177286.195	1177286.193	−0.002
Z (m)	4941629.347	4941629.336	−0.011	4941630.683	4941630.687	0.004
**Coordinates**	**Test Point 5**			**Test Point 6**		
	**GNSS Measured**	**Laser scanner Transformed**	**Differences**	**GNSS Measured**	**Laser Scanner Transformed**	**Differences**
X (m)	3835633.954	3835633.961	0.007	3835673.791	3835673.783	−0.008.
Y (m)	1177294.966	1177294.976	0.010	1177258.615	1177258.616	0.001
Z (m)	4941655.200	4941655.207	0.007	4941633.229	4941633.225	−0.004
